# Innate Immune Defense Mechanisms by Myeloid Cells That Hamper Cancer Immunotherapy

**DOI:** 10.3389/fimmu.2020.01395

**Published:** 2020-07-09

**Authors:** Els Lebegge, Sana M. Arnouk, Pauline M. R. Bardet, Máté Kiss, Geert Raes, Jo A. Van Ginderachter

**Affiliations:** ^1^Laboratory of Cellular and Molecular Immunology, Vrije Universiteit Brussel, Brussels, Belgium; ^2^Myeloid Cell Immunology Laboratory, VIB Center for Inflammation Research, Brussels, Belgium

**Keywords:** cancer immunotherapy, tumor-associated myeloid cells, tumor microenvironment, innate immune response, immune suppression, immunotherapy resistance

## Abstract

Over the past decade, cancer immunotherapy has been steering immune responses toward cancer cell eradication. However, these immunotherapeutic approaches are hampered by the tumor-promoting nature of myeloid cells, including monocytes, macrophages, and neutrophils. Despite the arsenal of defense strategies against foreign invaders, myeloid cells succumb to the instructions of an established tumor. Interestingly, the most primordial defense responses employed by myeloid cells against pathogens, such as complement activation, antibody-dependent cell cytotoxicity and phagocytosis, actually seem to favor cancer progression. In this review, we discuss how rudimentary defense mechanisms deployed by myeloid cells can promote tumor progression.

## Introduction

Immune cells abundantly infiltrate tumors, creating a complex environment mediated by repetitive cycles of antitumor responses and immune evasion (1). Myeloid innate immune cells, such as granulocytes, monocytes, macrophages and dendritic cells (DCs), play an important role in cancer-cell recognition, initiation of inflammation and antitumor responses (2). Chronic inflammation, however, can initiate tumorigenesis and can drive cancer progression in some cancer types (3, 4). Hence, myeloid cells play a dual role in cancer as they can initiate antitumor responses and communicate with cells of the adaptive immune system, but also promote local inflammation leading to chronic cancer-associated inflammation (5, 6).

In the tumor microenvironment, tumor-associated macrophages (TAMs) display an array of phenotypes beyond the M1/M2 paradigm, ranging from antitumoral to immunosuppressive, proangiogenic, immunomodulatory and tissue-remodeling phenotypes (7–9). The presence of TAMs in most solid tumors is correlated with poor prognosis and overall survival of patients (10). In addition to TAMs, solid tumors are also infiltrated by immunosuppressive, immature myeloid progenitor cells, commonly referred to as monocytic or polymorphonuclear myeloid-derived suppressor cells (M/PMN-MDSC) (11–13). Similarly, an increased infiltration of MDSCs has been associated with poor prognosis for a variety of cancer types (14). Neutrophils also contribute to tumor progression, yet establishing the difference between PMN-MDSCs and tumor-associated neutrophils (TAN) remains challenging (11, 15, 16). Although tumor-promoting functions have been attributed to other granulocytes, like eosinophils (17), basophils (18) and mast cells (19), further research is required to fully elucidate their role in cancer, as antitumoral roles have also been described (20, 21). Another myeloid population in the tumor microenvironment (TME) are DCs, that originate from different precursors and display various phenotypes, ranging from immunosuppressive monocyte-derived DCs (Mo-DCs) to immunocompetent cDC1 and cDC2 subsets (22). Altogether, the myeloid compartment in the TME is heterogenous and varies across tumor types, individuals and tumor stage (23). Nevertheless, the majority of scientific discoveries points toward a more tumor-supporting role for myeloid cells in the TME.

## Rudimentary Myeloid Defense Strategies as Tumor Promoters

**Figure 1:**
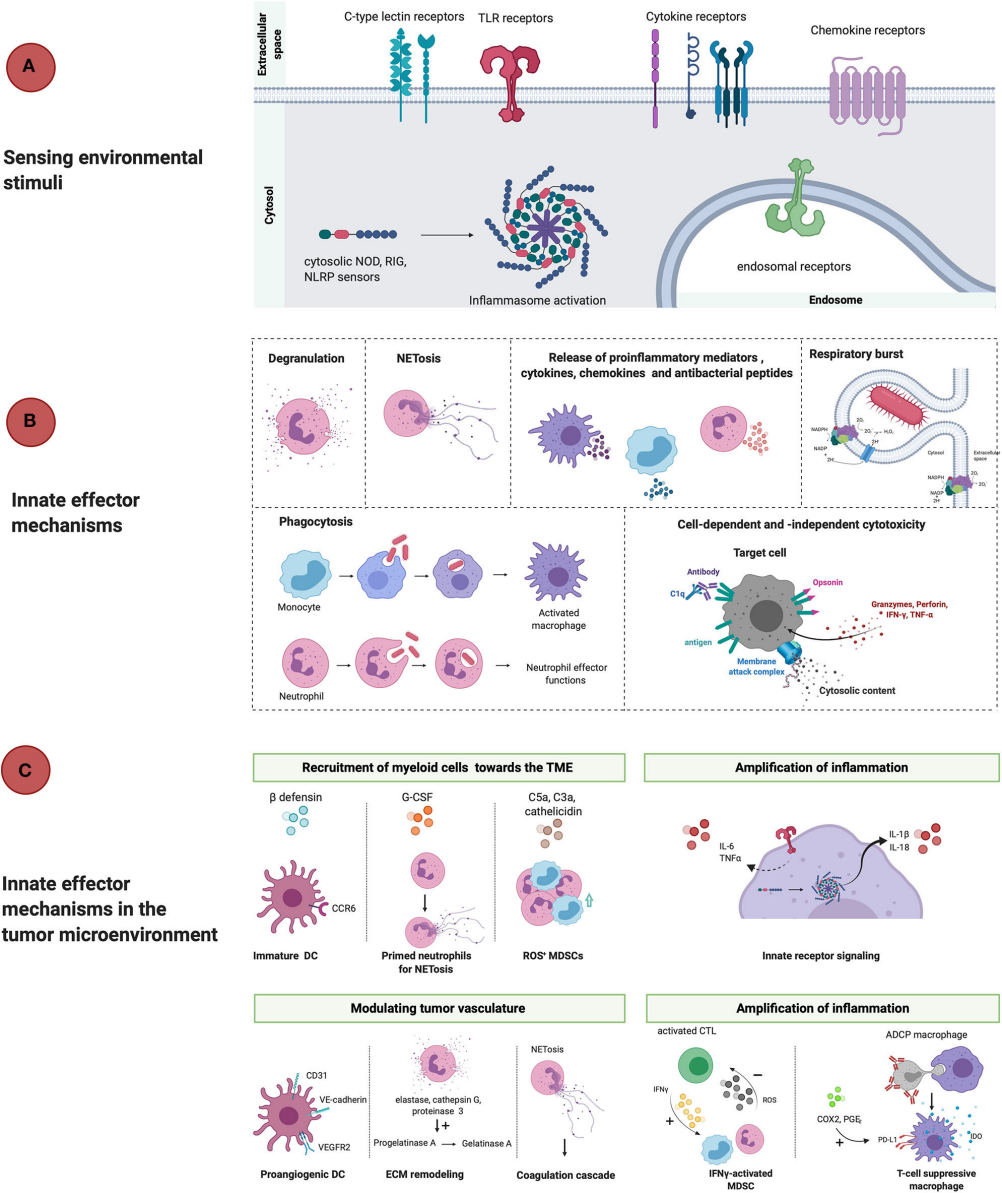
Linear representation of classical innate immunity in response to threats and in the TME. **(A)** PAMPs and DAMPs are recognized by surface-expressed, endosomal and cytosolic pattern recognition receptors (TLR, CLR, cytokine, chemokine receptors, NLRP3) which results in phenotypical changes that counteract ongoing threats or tissue damage. **(B)** Effector mechanisms that take place during inflammation are degranulation, NETosis, release of proinflammatory mediators, respiratory burst, phagocytosis and cell-dependent and -independent cytotoxicity. The net result is the recruitment of immunocompetent cells that mount an inflammatory reaction and potentially resolve the infection. **(C)** However, in the tumor microenvironment innate myeloid cells promote tumor progression through active recruitment to the TME in response to ß-defensins, cathelicidin, G-CSF, complement factors and chemokines. Once arrived in the TME, myeloid cells are activated and release proinflammatory mediators, which empowers tumor-associated inflammation. Activation of myeloid cells also allows for remodeling of the tissue vasculature and extracellular matrix, which also allows for cancer-cell invasion and metastasis. Furthermore, myeloid cells contribute to immunosuppression once activated by for example, upregulation of PD-L1 and IDO release during antibody-dependent phagocytosis of target cells or stimulatory cytokines (IFNγ). DC, dendritic cell; ECM, extracellular matrix; VEGFR2, vascular endothelial growth factor receptor 2; IFNγ, interferon gamma; ROS, reactive oxygen species; MDSC, myeloid-derived suppressor cell; ADCP, antibody-dependent cell-mediated phagocytosis; IDO, indoleamine 2,3-dioxygenase; COX2, cyclooxygenase 2; PGE2, prostaglandin E2; TNFα, tumor necrosis factor alpha; G-CSF, granulocyte colony stimulating factor; NOD, nucleotide-binding oligomerization domain; RIG, retinoic acid-inducible gene; NLRP, nucleotide-binding oligomerization domain; leucine-rich repeat and pyrin domain containing.

**Figure 2:**
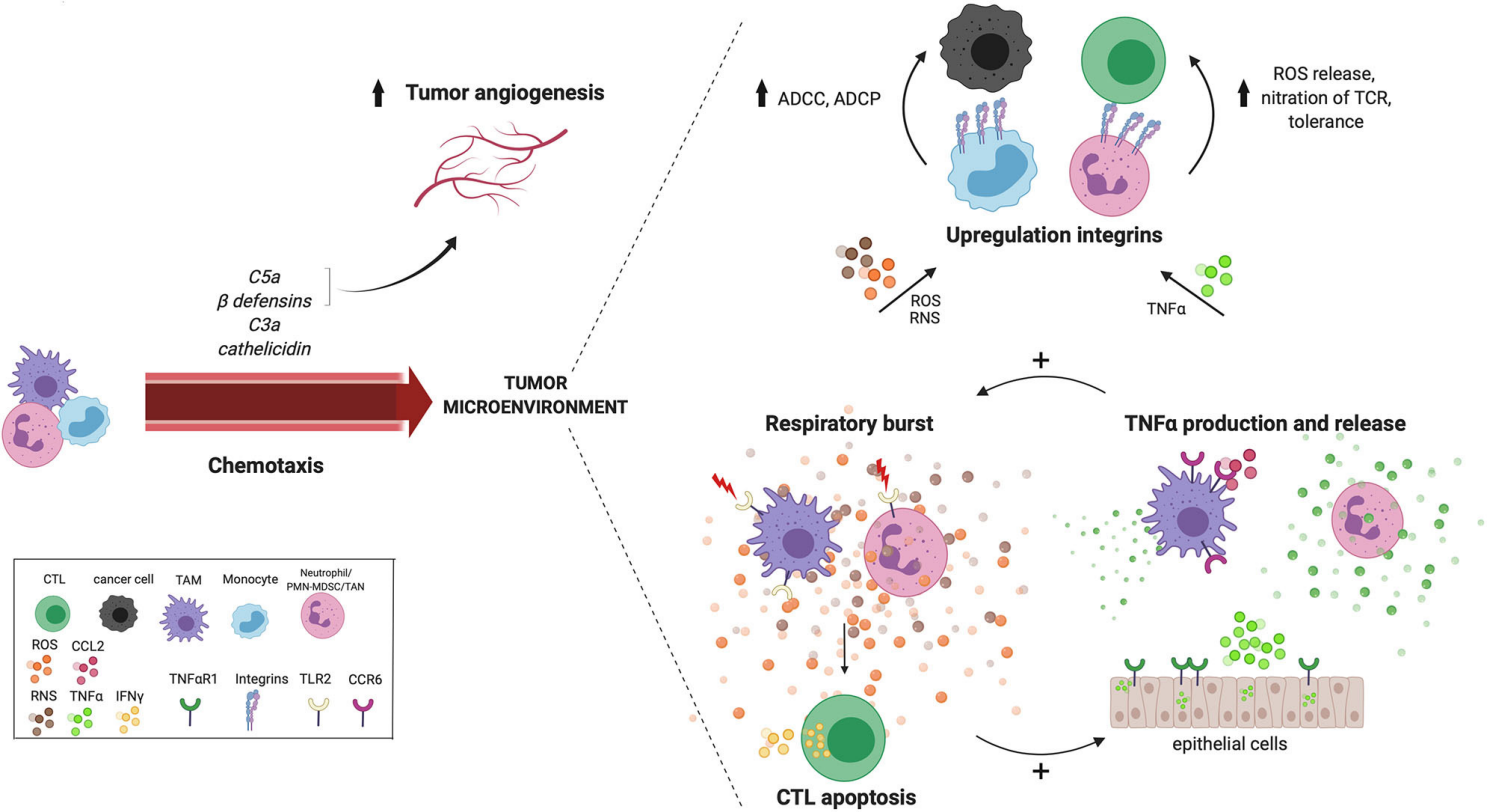
Cross talk between reoccurring innate effector mechanisms in the TME. Tumor-derived chemokines that are produced as a result of innate effector mechanisms including C5a, C3a, cathelicidin and ß-defensin, recruit myeloid cells to the TME. Tissue vasculature during chronic inflammation is maintained by complement anaphylatoxin C5a and beta-defensins. Anaphylatoxin C5a also recruits MDSCs with increased ROS and NRS production in the TME. Many innate pathways converge at the production of ROS and NOS in the TME. For example, TLR2 signaling increases the antigen presenting capacity of TAMs, which activates CTLs resulting in IFNγ release and subsequent ROS and NO release by TAMs. Neutrophil-derived ROS induces CTL apoptosis, while hydrogen peroxide released by TAMs, induces the expression of TNFα and TNFαR1 in surrounding epithelial cells. A positive feedback loop seems to exist between the respiratory burst and TNFα release, creating a potential cross talk between TAMs, neutrophils and epithelial cells in the TME. Furthermore, both ROS and TNFα also increases the expression of integrins, which increases cell-cell contact and facilitates cell-mediated killing via ADCC and ADCP either by performed by monocytes to kill cancer cells, or by MDSCs to suppress CTLs. ADCC, antibody-dependent cell-mediated cytotoxicity; MDSC, myeloid-derived suppressor cell; ADCP, antibody-dependent cell-mediated phagocytosis; ROS, reactive oxygen species; RNS, reactive nitrogen species; TNFα, tumor necrosis factor alpha; TNFαR1, tumor necrosis factor alpha receptor 1; CTL, cytotoxic T lymphocyte; IFNγ, interferon gamma; TCR, T-cell receptor; TAM, tumor-associated macrophage; CTL, cytotoxic T lymphocyte; TME, tumor microenvironment; TLR2, Toll-like receptor 2; TAM, tumor-associated macrophage.

The innate immune response by myeloid cells occurs as a succession of events starting at signaling through cytosolic or surface PRRs, followed by effector responses including the release of cytokines, reactive oxygen species (ROS), reactive nitrogen species (RNS), antibacterial peptides and degranulation (Figure 1). PRR on myeloid cells can be triggered by pathogen-associated molecular patterns (PAMPs) or danger-associated molecular patterns (DAMPs), but also indirectly by secondary mechanisms such as complement activation and circulating antibodies (Abs), resulting in cytolytic and phagocytic effector mechanisms. Pathogen clearance is mediated by mechanisms such as phagocytosis, respiratory burst with the production of ROS and RNS and release of bacteriostatic peptides, but also through the cell-extrinsic initiation of inflammation via the release of proinflammatory cytokines and chemokines (24). However, this succession of events does not always appear to be a linear cascade, as feedforward loops and interactions exist between different effector mechanisms (Figure 2). Yet, even such early, innate effector mechanisms performed by myeloid cells surprisingly seem capable of promoting tumor progression.

### Pathogen and Tissue Damage Recognition Mechanisms as Tumor Promoters

Toll-like receptors (TLRs), C-type lectin receptors (CLRs), the retinoic acid-inducible gene (RIG)-I-like receptors (RLRs) and NOD-like receptors (NLRs) are PRR families expressed by macrophages and DCs, but also by non-immune cells, such as endothelial cells and fibroblasts (25). Based on current literature, it seems that PRR signaling can both contribute to cancer progression and is capable of steering antitumor responses. Here, we will focus on the tumor-promoting role of PRRs, where TLR signaling and inflammasome activation will serve as an example to demonstrate the effect of PRR signaling in tumor-infiltrating myeloid cells.

In response to the tumor-derived proteoglycan versican V1, TLR2- and TLR6-signaling in TAMs induces the expression of cathelicidin (hCAP18/LL-37), an antimicrobial peptide which in turn promotes the proliferation of human ovarian cancer cells *in vitro* (26). When a TLR2-agonist, lipoprotein Pam2CSK4, is administered intravenously, TLR2-expressing PMN-MDSCs accumulate and proliferate systemically in EG7 lymphoma-bearing mice (27). Moreover, Pam2CSK4-mediated TLR2 signaling promotes the survival of M-MDSCs and mediates the differentiation of M-MDSCs into macrophages. These macrophages are capable of presenting tumor antigens to CTLs, resulting in interferon gamma (IFNγ) release upon T-cell activation and the subsequent expression of inducible nitric oxide synthase (iNOS) and nitric oxide (NO) release by surrounding macrophages, which in turn leads to T-cell suppression (28). In the presence of bacterial lipopolysaccharides in the colonic lumen, TLR4 signaling in TAMs promotes chronic inflammation through increased production of cyclo-oxygenase 2 (COX2) and prostaglandin E_2_ (PGE_2_) (29). Damage-associated high mobility group box-1 protein (HMGB1), released from necrotic keratinocytes in the skin upon irradiation, interacts with TLR4 on bone marrow-derived immune cells (30). The resulting signaling facilitates papilloma progression through an increase in the recruitment of proinflammatory immune cells (30). Moreover, HMBG1-mediated TLR4 signaling causes an increased infiltration of radiation-resistant cells upon radiotherapy.

Upon intracellular PAMP or DAMP recognition by cytosolic sensors like NLRP3, inflammasomes are assembled, which results in the release of the proinflammatory cytokines IL-1ß and IL-18 and leads to a proinflammatory form of cell death, also referred to as pyroptosis (31). In different murine tumor models, NLRP3 plays a role in the migration of MDSCs to the TME, where MDSCs suppress antitumor CTL responses independent of NLRP3 and induce unresponsiveness to DC vaccination (32). The role of inflammasome activation in tumor progression is also demonstrated in obese mice, where obesity-associated NLRC4 inflammasome activation in tumor-infiltrating myeloid cells promotes breast cancer progression (33). Importantly, the release or administration of PRR agonists may give rise to therapy resistance in patients that underwent radiotherapy (34), chemotherapy (35, 36) or cancer vaccination (32). For example, myeloid Gr1-negative cells accumulate in murine B16 melanoma and CT26 colon adenocarcinoma tumors after local irradiation, where mitochondrial DNA of dead, irradiated cancer cells induces TLR9 signaling, which mediates revascularization and immune evasion in an interleukin (IL)-6- and STAT3-dependent manner (34, 37). Paclitaxel-induced TLR4 signaling in murine and human breast cancer cells results in the production of the proinflammatory cytokines IL-1ß and IL-6, which promotes the expansion of MDSCs in the bone marrow and spleen as well as their recruitment to the TME (36). In response to gemcitabine and 5-fluorouracil chemotherapy, cathepsin B is released in the cytosol of MDSCs which induces NLRP3-dependent IL-1ß release (35). In return, IL-1ß drives the polarization of CD4+ T cells into Th17 cells that promote tumor angiogenesis in the TME, which hampers the antitumor response of gemcitabine and 5-fluorouracil.

Altogether, it seems that the tumor microenvironment can be a source of PRR agonists, stimulating PRR signaling in myeloid cells that in turn perform tumor-promoting functions. Alternatively, PRR signaling can also directly affect cancer cells. TLR4 expression and signaling in gastric cancer cells results in mitochondrial ROS production, which induces secondary signaling cascades in response to oxidative stress that may regulate cancer-cell survival (38). TLR4 signaling in colorectal cancer and breast cancer cells promotes invasion and metastasis of these cells (36, 39). Therefore, PRR signaling is not strictly a myeloid cell-restricted, tumor-promoting mechanism.

### Release of Proinflammatory Mediators as Tumor Promoters

A common downstream effect of PRR signaling is the release of proinflammatory cytokines, like IL-12, IL-6, IL-1β and tumor necrosis factor alpha (TNFα). In the TME, cytokines like IL-10 and transforming growth factor beta (TGF-ß) play an important role in suppressing antitumor responses, so it is within expectation that strongly opposing, proinflammatory mediators would be capable of eliciting and sustaining antitumor responses. However, a number of key proinflammatory cytokines, such as IL-1β and IL-6, have been reported to promote tumor progression through the mobilization of MDSCs (40, 41), the contribution to chronic inflammation (40, 42) and the stimulation of angiogenesis (43, 44). For example, in murine models of pancreatic ductal adenocarcinoma, neutralization of tumor-derived IL-1β enhances CTL-infiltration and ameliorates the response to anti-PD-1 immune checkpoint blockade (45). In accordance, IL-1β-blockade synergizes with anti-PD-1 immune checkpoint blockade in 4T1 breast cancers by restoring the cytotoxic capacity of CTLs without inducing systemic inflammation (46).

Other proinflammatory cytokines, such as TNFα and IFNγ, seem to have an ambiguous effect on cancer progression. For example, neutrophil-derived TNFα promotes the production of NO in an autocrine manner, which in turn induces apoptosis of non-activated CTLs in murine models of thoracic malignancies (47). Subcutaneous *Tnfr1*-deficient fibrosarcoma FB61 tumors are rejected in *Tnfr*-deficient mice, while tumor growth is reestablished via an adoptive transfer of *Tnfr1*-expressing MDSCs. Mechanistically, MDSCs of *Tnfr*-deficient mice displayed increased caspase-8 cleavage which induces apoptosis, and lower levels of c-FLIP, a natural caspase-8 inhibitor, which causes reduced accumulation of MDSCs in the TME along with a reduced tumor-suppressive capacity (48). These data suggest that endogenous and persistent TNFR signaling promotes tumor growth by maintaining survival of MDSCs (48). In accordance, a study by Sade-Feltman et al. demonstrated that TNFα is required to maintain the immature and immunosuppressive phenotype of MDSCs (49). Hence, TNFα blockade using Etanercept, a biological compound composed of the extracellular domain of TNFR fused to an IgG1 Fc fragment, restores NK-cell cytotoxicity and T-cell proliferation, reduces splenic MDSC accumulation and enhances the maturation of MDSC into CD11b^+^CD11c^+^ and CD11b^+^ F4/80^+^ cells (49). In addition, TNFα induced upon anti-PD-1 immune checkpoint blockade, increases PD-L1 and TIM-3 expression on tumor-infiltrating T cells and promotes their cell death upon TNFα binding to TNFRs (50). TNFα blockade increases the infiltration of tumor-specific CTLs, reduces the proliferation of immunosuppressive, regulatory T cells (Tregs) and minimizes toxicity of immune checkpoint blockade (51–53). These tumor-promoting effects of TNFα in the TME are in contrast to its inhibition of breast cancer-cell proliferation by blocking the G1/S phase transition of the cell cycle (54). Furthermore, TNFα may hamper the polarization and differentiation of monocytes into M2-like TAMs, instead steering the macrophage phenotype toward an anti-tumoral M1-like TAM in the TME (55). Altogether, TNFα also carries the potential to mount antitumoral responses in cancer therapy, as described elsewhere (56).

The role of the proinflammatory cytokine IFNγ in tumor progression appears to be concentration- (57, 58) and context-dependent (28, 59). He et al. demonstrated that, at low local levels, IFNγ promotes tumor progression of several murine tumor models, including hepatic, mammary and skin cancer, through increased gene expression of *Cd274* (PD-L1), *Ctla4* and *Foxp3*, whereas at higher levels, IFNγ reduces the gene expression of *Foxp3* and co-inhibitory molecules (58). If either TNFα or IFNγ signaling in tumor-infiltrating CD4^+^ T cells is absent upon antigen recognition, tumor progression is stimulated, whereas combined TNFα and IFNγ signaling in CD4^+^ T cells prevents tumor angiogenesis and tumor-cell proliferation (59). Hence, cytokines like IFNγ and TNFα can play dual roles in cancer progression and the internal complexity of combined receptor signaling strongly affects antitumor responses (59).

Besides cytokines, other inflammatory mediators influence tumor progression. Indeed, proinflammatory enzymes and products of the prostaglandin production pathway, including COX2 and PGE_2_, have been associated with enhanced tumor progression, as they induce the expression of PD-L1 on macrophages and MDSCs (60). A tumor-promoting feedback loop has been discovered between MDSCs, colorectal cancer cells and T cells, that all release PGE_2_ and express receptor-interacting protein kinase 3 (RIPK3) (61). PGE_2_-induced RIPK3 signaling in MDSCs results in the expression of COX2 that catalyzes PGE_2_ synthesis, which is then released in the TME. PGE_2_ promotes proliferation of cancer cells and suppresses T-cell activation through RIPK3 signaling. Macrophage-derived IL-1β induces ROS-dependent COX2 production and activity in breast cancer cells, leading to PGE_2_ release *in vitro* (62). Culturing blood-derived monocytes with PGE_2_ induces the expression of COX2, which inhibits differentiation of monocytes into monocyte-derived DCs. Instead, the expression of indoleamine 2,3-dioxygenase (IDO), IL-4 receptor, iNOS and IL-10 is upregulated and drives the suppressive phenotype of M-MDSCs *in vitro* (63). Hence, PGE_2_ contributes to polarizing the phenotype of myeloid cells in the TME.

In conclusion, two trends are observed regarding proinflammatory cytokines or mediators; (1) either their role in cancer progression is generally protumoral, such as IL-6, IL-1β, or PGE_2_, or (2) their function in cancer progression is ambiguous, such as for TNFα and IFNγ. The severity of inflammation may play an important role here; to a certain extent, proinflammatory mediators are required to stimulate anti-tumoral T-cell responses, whereas prolonged exposure or exposure to high levels of inflammatory mediators can lead to unresponsiveness. In addition, it is not clear whether cancer cells or myeloid cells initiate the expression and release of tumor-promoting inflammatory mediators in the TME.

### Respiratory Burst as Tumor Promoter

Upon PAMP recognition through PRR signaling, neutrophils and macrophages engulf pathogens via phagocytosis, which activates phagosome- and surface membrane-bound NADPH oxidase, resulting in the production of superoxide (${\text{O}}_{2}^{-}$) and derivatives, hydrogen peroxide (H_2_O_2_) and hypochlorous acid (HOCl), through downstream processing by superoxide dismutase (SOD) and myeloperoxidase (MPO) (64–66). The release of ROS in phagosomes and the extracellular space is referred to as the respiratory burst, which is a primary antimicrobial and antifungal defense mechanism deployed by phagocytes (64). MDSCs are a major source of ROS in the TME, where ROS and peroxynitrite (${\text{HNO}}_{3}^{-}$) abrogate antigen recognition by CTLs and instead induce tolerance (67, 68). This depends on direct contact between T cells and MDSCs, mediated by the integrins CD11b, CD18, and CD29 (68). Mechanistically, nitration and oxidation of amino acids in the T-cell receptor (TCR) and CD8 co-receptor molecules prevents interaction with major histocompatibility complex (MHC) molecules, which in turn induces tolerance (67). Constitutive upregulation of STAT3 in MDSCs directly regulates the expression of NOX2 components necessary for the formation of the NADPH protein complex, which is followed by a subsequent increase in production and release of ROS (69). MDSCs are unable to suppress T cells in the absence of NOX2 activity, and instead differentiate into mature macrophages and DCs (69). ROS also mediate the polarization of macrophages, as inhibition of ${\text{O}}_{2}^{-}$ impedes the differentiation of monocytes into M2 macrophages while differentiation into M1 macrophages remains unaltered (70, 71). Thus, while ROS production in MDSCs maintains their immature phenotype, MDSC-derived ROS in the TME mediates the differentiation of tumor-infiltrating monocytes. Furthermore, H_2_O_2_ released by macrophages and neutrophils induces the expression of *Tnfa* and *Tnfr1* in epithelial cells, that in turn release TNFα leading to the upregulation of other proinflammatory and angiogenic factors, hence, sustaining tumor progression in a paracrine loop (72). Aside from myeloid-derived ROS, Xia et al. demonstrated that ROS can also be produced by cancer cells themselves. They showed that ROS production by ovarian cancer cells promotes angiogenesis and tumor growth through *in vivo* transcriptional activation of *Vegf* and *Hif1a* (72, 73). The above-mentioned studies provide evidence for the protumoral role of ROS in tumor progression, by suppressing T-cell responses, supporting angiogenesis and maintaining the phenotypical identity of MDSCs, regardless of the strong pathogen-killing potential of the respiratory burst in mature myeloid cells.

### Release of Antibacterial Peptides as Tumor Promoter

In addition to ROS, myeloid cells release a vast array of antimicrobial peptides such as defensins and cathelicidins, representing two major families of mammalian antibacterial peptides. In leukocytes, α- and ß-defensins are stored in cytoplasmic granules that fuse with the phagosome upon microbe phagocytosis, while epithelial cells can secrete defensins to maintain their barrier integrity (74). Yang et al. demonstrated that ß-defensins act as a chemoattractant for immature DCs and memory T cells by binding chemokine receptor CCR6, which bridges the innate recognition of microbes and the initiation of an adaptive immune response (75). As such, it is not surprising that in a similar fashion immature DCs are recruited to the TME in response to tumor-derived ß-defensins. Indeed, Conejo-Garcia et al. discovered a subset of immature DCs, that is recruited to murine and human ovarian tumors in response to ß-defensins through CCR6 signaling and that acquires epithelial features, including surface expression of CD31 and VE-cadherin. These cells support vasculogenesis in a VEGFR-2-dependent manner which leads to enhanced tumor progression (76). CCR6 signaling also promotes murine transplantable colon cancer by recruiting macrophages to the TME through a CCL2-CCR6 axis, which results in the release of IL-1β, IL-6, and TNFα, further enhancing tumor progression (77).

Holterman et al. reported that α-defensins overexpressed by cancer cells, stimulate the proliferation and migration of bladder cancer cell lines *in vitro*, most-likely in an autocrine and calcium-dependent manner (78). Similarly, Xu et al. showed that human ß-defensin 3 promoted *in vitro* proliferation, migration and invasion of cervical cancer through the NF-κB signaling pathway, demonstrating that cancer cells are also able to release defensins (79). It is important to note that defensin-secreting cancer cells are of epithelial origin, since epithelial cells are known to secrete defensins as part of their barrier function. In addition, it should be remarked that the role of defensins in tumor progression also seems ambiguous and may vary according to the cancer type or defensin molecule, as several studies showed a potential antitumoral role of defensins in cancer (80, 81).

The release of cathelicidins, human LL-37 and murine CRAMP, in the TME has been described in several studies, whereby macrophages and neutrophils are the main sources. Li et al. demonstrated that CD68^+^ macrophages in tumor tissue of colorectal cancer patients stained positive for cathelicidin, whereas weak to unmeasurable signal was picked up for cathelicidins in colon epithelial cells (82). The importance of cathelicidins in tumor progression was demonstrated by a slower tumor growth in Lewis lung carcinoma-bearing, cathelicidin-deficient mice, along with a reduced infiltration of myeloid cells (83). These studies suggest that cathelicidins are chemoattractants that recruit myeloid cells to the TME (84), where, in turn, myeloid-derived cathelicidins directly enhance cancer-cell proliferation, creating a self-sustaining loop of cathelicidin production. In contrast, antitumoral roles of cathelicidins, independent of myeloid cells, have also been described. For example, cathelicidins could be involved in potentiating the cytotoxic capacity of tumor-infiltrating NK cells (85) and impairing the tumor-supportive role of cancer-associated fibroblasts (CAFs) in colon cancer (86).

### Neutrophil Degranulation as Tumor Promoter

Neutrophils carry heterogenous primary, secondary and tertiary granules that contain different enzymes and modulatory proteins, such as elastase, gelatinase, MPO, cathepsins, ficolin-1, and lactoferrin (87). Neutrophil degranulation occurs in a calcium-dependent manner in response to proinflammatory mediators like TNFα (88), lipopolysaccharides (LPS) (89) and IL-8 (90). The majority of neutrophil-derived granule contents promote tumor progression, such as elastase, cathepsin D, cathepsin B, and proteinase 3.

Neutrophil-derived elastase hydrolyses insulin receptor substrate-1 (IRS1) in the cytosol of lung cancer cells, leading to an altered regulation of phosphoinositide 3-kinase (PI3K). IRS1 degradation indirectly increases the interaction between the p85 protein of PI3K and platelet-derived growth factor receptor (PDGFR), which enhances cancer-cell proliferation through signaling downstream of the PDGFR (91). Elastase released by PMN-MDSCs in lymphangioleiomyomatosis patients, a condition where estrogen-sensitive metastatic tumors grow in the lungs, stimulate the proliferation, migration and invasion of these tumor cells *in vitro* (92). Cathepsin-D stimulates cancer-cell proliferation as well, but also stimulates tumor angiogenesis and could protect cancer cells from apoptosis (93). Hepsin, a transmembrane serine protease involved in cell motility and shape, is degraded by the proteasome through cathepsin D-stimulated ubiquitination (94). By downregulating hepsin, cathepsin D contributes to enhanced migration and invasion of breast cancer. Cathepsin B cleaves cell cycle inhibitor p27^Kip1^ in the lysosomes of colorectal cancer cells, which contributes to tumorigenicity and metastasis of colorectal cancer cells (95). Extracellular matrix (ECM) and intracellular collagen IV can be degraded by cathepsin B, stimulating tumor invasion, metastasis, and the formation of vessel-like structures *in vivo* (96).

Although proteinase 3 can be secreted by myeloid cells, neutrophils carry a membrane-bound proteinase 3 that seems to play a role in cellular interactions. Neutrophils in acute myeloid leukemia inhibit T-cell proliferation in a contact-dependent manner. Antibody-based blockade of membrane-bound proteinase 3 on the surface of neutrophils partially restores proliferation of CD4^+^ and CD8^+^ T cells (97). The resulting signaling cascade caused by the interaction between membrane-bound proteinase 3 on neutrophils and receptor for advanced glycation end-products (RAGE) on prostate cancer cells promotes tumor-cell migration and metastasis to the bone barrow, independent of the proteolytic activity of proteinase 3 (98). Combined efforts of neutrophil elastase, cathepsin G and proteinase 3 activate progelatinase A, that degrades the extracellular matrix followed by the subsequent release of growth factors, tumor-cell invasion and angiogenesis in the TME (99). In conclusion, the majority of enzymes released or upregulated upon neutrophil degranulation can remodel the extracellular matrix, which stimulates tumor-cell invasion, metastasis and tumor growth, but also promotes tumor angiogenesis.

### Neutrophil Extracellular Trap Formation (NETosis) as Tumor Promoter

Neutrophil extracellular traps (NETs) are extracellular strands composed of granule content and nuclear fragments that entrap and kill bacteria through granule proteases and DNA histones (100, 101). Various studies have demonstrated that the formation of NETs is ROS-dependent (64, 102, 103), but can also occur through CXCR2 signaling during chronic inflammation, and through TLR2 and C3 signaling (101, 104). A study unraveling the role of high sensitivity troponin T (hsTnT) plasma levels in the onset of ischemic stroke, revealed an unexpected high prevalence of cancer among patients with elevated hsTnT plasma levels in the post mortem analysis (105). In these patients, the elevated hsTnT plasma level was associated with an increased plasma level of NET-associated citrullinated histone H3, a marker for NETosis, as well as increased plasma levels of G-CSF and coagulation factors. This study demonstrates that NETosis can take place in cancer patients with elevated citrullinated histone H3 levels (105). In fact, tumor-derived G-CSF primes neutrophils to form NETs, which could also contribute to a systemic, prothrombic state in these cancer patients (105, 106).

Furthermore, a study by Miller-Ocuin et al. correlated circulating neutrophil DNA, resulting from NETosis, to the cancer stage of pancreatic ductal adenocarcinoma patients (107). They demonstrated that neutrophil DNA activates pancreatic stellate cells that support tumor progression, and propose that NET DNA acts as a DAMP capable of stimulating tumor progression (107). In patients that underwent major liver resection of metastatic colorectal cancer, in which ischemia and reperfusion is inevitable, NET formation was increased compared to cancer patients that underwent minor liver resection, in which ischemia and reperfusion is limited, demonstrating that surgery-induced stress promotes NET formation (108). These authors further demonstrated that NETs in the liver provide an anchoring site for circulating cancer cells, that supports metastases and cancer-cell growth after resection of the primary tumor. Hence, NETs may support tumor progression through various mechanisms.

Surrounding macrophages deal with the aftermath of NETosis by digesting cellular debris. Interestingly, M1-like macrophages have been shown to release uncoiled or uncondensed DNA upon interaction with NETs *in vitro*, suggesting a possible contribution to NETosis through their own form of extracellular trap formation (METosis) (109). It remains to be seen whether such a mechanism contributes to the tumor-promoting effects of macrophages.

### Complement Activation as Tumor Promoter

Complement is an innate defense mechanism that detects and eliminates pathogens from the circulation and tissues, clears cellular debris and stimulates adaptive immunity.

Complement activation through the classical, alternative or lectin-mediated pathway ultimately results in the formation of a cytolytic membrane attack complex (MAC) in the membrane of target cells or microorganisms (110) and the production of anaphylatoxins C3a and C5a (111). Anaphylatoxins can be involved in T-cell homeostasis (112, 113) and in the recruitment of granulocytes (114–116), monocytes (117) and DCs (118) to the site of inflammation through chemotaxis via C3a and C5a receptors (C3aR, C5aR).

Aside from anaphylatoxin production during complement activation, opsonin C3b and its cleavage products (iC3b, C3c, C3d) are deposited on the surface of target cells or microorganisms, when C3 is cleaved by C3 convertase (119). Myeloid cells express complement receptors that bind C3-derivatives, leading to phagocytosis, cell-cell adhesion and adhesion to the extracellular matrix (120). Complement can also steer the adaptive immunity by activating B and T cells through combined engagement of complement receptors and the B-cell receptor or TCR, respectively (121).

**Figure 3:**
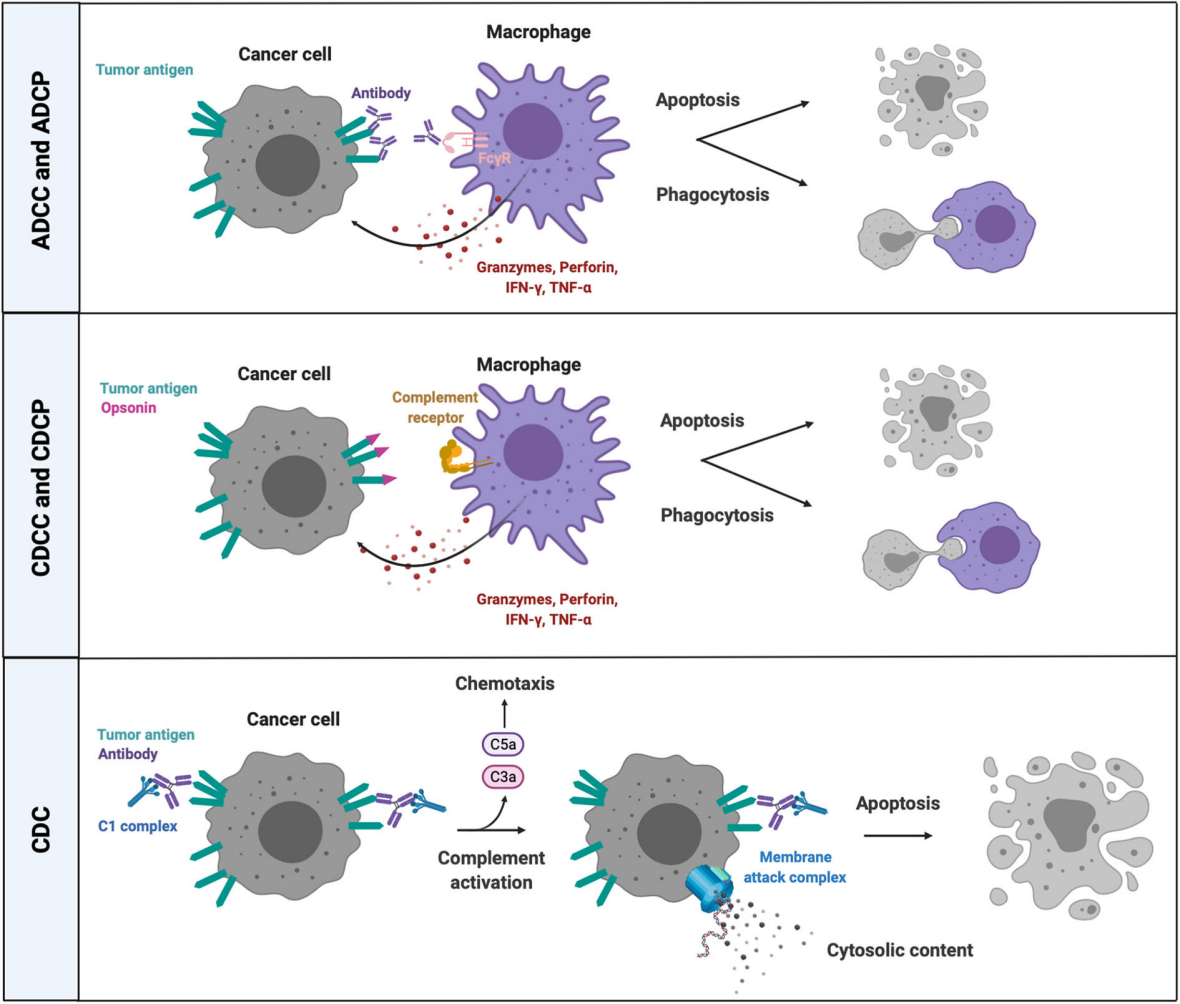
Cell-dependent and -independent effector mechanisms of complement activation and FcR-mediated killing. Complement factor- and antibody-opsonized cancer cells can be eliminated through cell-dependent and cell-independent effector mechanisms. CRs and FcRs on phagocytes bind opsonins and antibodies, respectively, on the surface of targeted cancer cells, followed by phagocytosis and/or release of lytic enzymes (granzyme B, perforins) and proinflammatory mediators (TNFα, IFNγ). The classical pathway of complement activation mediates a cell-independent form of lytic cell death by introducing a MAC in the membrane of antibody opsonized target cells that are recognized by complement C1 complex. ADCC, antibody-dependent cell-mediated cytotoxicity; ADCP, antibody-dependent cell-mediated phagocytosis; CDC, complement-dependent cytotoxicity; CDCC, complement-dependent cell-mediated cytotoxicity; CDCP, complement-dependent cell-mediated phagocytosis; CRs, complement receptors; IFNγ, interferon gamma; TNFα, tumor necrosis factor alpha; C5a, complement factor 5a; C3a, complement factor C3a; FcγR, crystallizable fragment receptor gamma; C1, complement factor.

Overall, the resulting effector mechanisms of complement activation are (1) cell-mediated phagocytosis (complement-dependent cellular phagocytosis or CDCP) and (2) cytotoxicity (complement-dependent cellular cytotoxicity or CDCC), initiated by the interaction between opsonized target cells or microbes and CR-expressing myeloid cells, as well as (3) complement-dependent cytotoxicity (CDC) through the formation of the MAC in the membrane of target cells or microorganisms, and (4) the recognition and clearance of dying cells (122) (Figure 3). However, distinguishing the different effector mechanisms that contribute to cancer-cell eradication as a result of complement activation remains challenging up to now. Furthermore, complement-induced cytolytic effector mechanisms on the surface of host cells is prevented through the expression of complement regulatory proteins (CRPs), such as CD46, CD55, CD59 and factor H. Several cancer types overexpress CRPs and make use of this defense mechanism against complement-induced cytolysis (123–125), whereas downregulation or blockade of CRPs sensitizes cancer cells to complement- and antibody-mediated cytotoxicity (126, 127).

#### Complement Anaphylatoxins as Tumor Promoters

Complement activation has been reported to promote tumor progression through the recruitment of immune suppressive macrophages, MDSCs and neutrophils, while on the other hand, there are also reports of its capacity to stimulate antitumoral T-cell responses and the recruitment of NK cells (128–130). Recruitment of MDSCs in response to anaphylatoxins has been demonstrated in several studies (130–132). Markiewski et al. revealed that aside from increased recruitment of MDSCs to the tumor in response to C5a, the latter also enhances the production of ROS and RNS in MDSCs via C5aR signaling (132). As mentioned earlier, ROS and RNS release by MDSCs in the TME abrogates antigen recognition by CTLs and instead induces tolerance (67, 68). Moreover, C5a is also implicated in the formation of new blood vessels. Corrales et al. demonstrated that human umbilical vein endothelial cells treated with C5a form vessel-like structures. They further elaborated on the vessel-like structures in a murine 3LL lung cancer model, where they showed that the number of newly formed microvessels in the tumor is reduced upon C5aR antagonism (133). While the role of C5a in cancer progression has been extensively studied, less is known about the implication of C3a in cancer. In the absence of C3aR signaling, murine B16 melanoma tumor growth is reduced, along with an increased tumor infiltration of CD4^+^ T cells and neutrophils (134). Similar results were observed in orthotopic mouse models of lung cancer (CMT167, LLC), where flow cytometry and immunohistochemistry analysis revealed an increased abundance of activated CD4^+^ and CD8^+^ T cells in tumors grown in C3-deficient mice (135). Interestingly, depletion of CD4^+^ T cells, but not CD8^+^ T cells, restored tumor growth in C3-deficient mice.

Tumor-infiltrating macrophages and neutrophils also carry the potential to suppress the detrimental effects of complement activation through IL-1ß-induced expression of pentraxin 3 (PTX3) (136). Surface-expressed PTX3 recruits complement factor H that inhibits the C3 cleavage upstream of the complement cascade and prevents complement-induced inflammation and recruitment of immunosuppressive myeloid cells to the TME. However, *Ptx3* is epigenetically silenced at the gene level in murine and human colorectal cancer through hypermethylation (136). Altogether, the above-mentioned studies provide evidence for the role of complement in cancer that seems to promote tumor progression by recruiting MDSCs to the tumor, reducing the infiltration of activated CD4^+^ T cells and stimulating new vessel formation. The effect of anaphylatoxins on tumor-infiltrating CTLs remains unresolved, whereas several studies highlight the importance of CD4^+^ T cells in response to anaphylatoxins.

#### Complement in Cancer Immunotherapy

Despite the intrinsic protumoral functions of complement in cancer, it should not be forgotten that complement can be useful in the context of antibody-mediated cancer immunotherapy. Indeed, the classical pathway of complement activation, initiated by antibody-opsonized target cells, is one of the effector mechanisms of therapeutic monoclonal antibodies (mAb) (137, 138). This was demonstrated in a study by Lee et al., who designed therapeutic mAbs capable of discerning complement-mediated and Fc receptor (FcR)-mediated killing mechanisms (139). Aglycosylated, anti-CD20 IgG1 mAb, engineered with a C1q-selective Fc-part that does not bind FcRs, demonstrated similar potency in clearing CD20^+^ Raji and Ramos lymphoblastic cells compared to antibodies that rely on FcR-mediated functions (139). Along the same line, the therapeutic anti-CD20 mAb Rituximab at least partially relies on the classical complement activation pathway for destruction of neoplastic CD20^+^ B cells (140). However, the release of proinflammatory mediators (IL-6, TNFα) and degranulation by granulocytes in response to complement anaphylatoxins contribute to the toxic side effects of anti-CD20 therapy, such as fever, dyspnea, chills and flushes (141). Similarly, the *in vivo* effector functions of Cetuximab, an anti-EGFR mAb, have been attributed to complement activation in several murine models of non-small cell lung carcinoma (142). However, it should be remarked that the efficacy of mAb-mediated complement activation is likely to be cancer type-dependent and may be influenced by the characteristics of the cancer cells and/or factors present in the tumor microenvironment.

Moreover, the efficacy of antibody-based therapy, that relies on the cytotoxic effector mechanisms of complement and FcR-mediated cytotoxicity, is restricted by the limited availability of suitable antigens for therapeutic targeting. In addition, the dual role of complement in cancer must be taken into account when using complement as an effector mechanism of antibody-based therapy. It appears that complement can promote tumor growth through high C5a concentrations, sublytic MACs levels and high CRP levels on the surface of cancer cells, while intermediate concentrations of C5a, increased MAC formation in the membrane of cancer cells and low surface expression of CRPs could eliminate cancer cells (129, 143). Future therapeutic strategies should take this delicate balance between tumor promotion and tumor eradication into account.

### FcR-Mediated Killing

When the Fc part of an antibody interacts with cognate surface-expressed FcRs, this may result in ADCC, ADCP, antigen presentation, degranulation and an altered cytokine production profile (Figure 3) (144). NK cells are thought to be the main effector cells of ADCC, yet studies have shown that antibody-based cellular destruction mechanisms can also take place in the absence of NK cells (145). The relevance for therapeutic mAbs is shown by mice deficient in the common gamma chain of the FcγR. These mice do not engage ADCC or ADCP in the presence of Trastuzumab and Rituximab (145, 146). Members of the mononuclear phagocyte system, including monocytes and macrophages, are responsible for the working mechanism of Rituximab (145). Indeed, CD20-targeted B-cell depletion seems to be dependent on FcγRI and FcγRIII expressed by monocytes and macrophages and is absent in colony stimulating factor 1-deficient mice, which lack tissue macrophage subsets (145). Biburger et al. discovered a murine subset of Ly6C^low^ non-classical monocytes capable of autoantibody-mediated platelet depletion and antibody-dependent B-cell depletion via ADCC and ADCP mediated by FcγRIV, a low affinity FcγR that is not expressed by NK cells or tissue-resident macrophages (147). Human CD16^+^ (FcγRI) monocytes similarly perform ADCC, almost as efficiently as NK cells. TNFα release by these CD16^+^ monocytes upregulates type 2 beta integrins (CD11a, CD11b), which facilitate the interaction between CD16^+^ monocytes and antibody-coated cancer cells (148). The number of murine B16 melanoma metastases in the lung of FcγRIIb-deficient mice significantly decreased when treated with a mAb targeting melanoma differentiation antigen gp75 (146). FcγRIIb is an inhibitory Fc receptor which is not expressed by NK cells. Therefore, an enhanced ADCC response cannot be attributed to increased NK-cell activation in FcγRIIb-deficient mice and is likely monocyte/macrophage-mediated. Moreover, a synergistic effect was observed when combining FcγRIIb deficiency and a therapeutic mAb against mouse and human HER2 (4D5, Trastuzumab) (146).

However, not all FcR-mediated effects are beneficial in the context of mAb-mediated therapy. For example, phagocytosis of antibody-opsonized cancer cells by TAMs was shown to activate the inflammasome AIM2, which results in the subsequent release of IL-1ß, hence increasing PD-L1 surface expression and cytosolic IDO production in TAMs (149). As a result, TAMs that underwent ADCP display an immunosuppressive phenotype, which is relieved upon PD-L1 and IDO blockade (149). Furthermore, *in vivo* imaging by Arlauckas et al. (150) demonstrated that PD-1-negative TAMs take up anti-PD-1 antibodies that were initially bound to PD-1^+^ CTLs, in an FcR-mediated way. Hence, TAMs could serve as a sink for anti-PD-1 antibodies and possibly also other mAbs, strongly diminishing the efficacy of mAb-dependent therapies such as immune checkpoint blockade (150).

## Entangled Network of Innate Responses

Innate immune responses are often regarded as the default first-line defense responses, that become less significant once a more complex, adaptive and antigen-directed response is initiated. With this review, we provide evidence for the detrimental effects of innate effector mechanisms performed by myeloid cells during cancer development and progression. Noteworthy, effector mechanisms that are initially deployed by innate myeloid cells, such as ROS production, release of inflammatory mediators and response to PRR signaling, can be adopted by cancer cells. However, contradicting literature studies are available on the role of several innate defense mechanisms in cancer, and this duality between tumor-promoting and -eradicating roles seems to be linked to the presence of persisting, tumor-associated inflammation. Inflammation is required to mount anti-tumor immune responses, while chronic tumor-associated inflammation promotes tumor progression. This duality can even be extended to the response of so called “hot tumors” and “cold tumors” to immunotherapy. Interestingly, mAb therapy targeting immune checkpoints seem to be effective in “hot tumors,” abundantly infiltrated by T cells, whereas “cold tumors” that lack proper T-cell responses remain largely unresponsive to mAb therapy (151). Cold tumors, however, are still infiltrated by myeloid cells, that create an immune suppressive environment, which impedes T-cell infiltration and tumor eradication. Therefore, innate defense strategies might play a more important role in cancers with an inflammatory nature or origin, for example in organs like the liver, stomach, lungs and skin due to alcohol abuse, *H. pylori* infection, tobacco and asbestos, UV irradiation and even obesity. In any case, due to the abundance of tumor-infiltrating myeloid cells in multiple solid tumor types, their effector mechanisms should be investigated in depth and exploited in cancer therapy, perhaps alongside T-cell stimulatory immunotherapy to improve therapy outcome.

## Author Contributions

EL has conceptualized, written, reviewed, and edited the content of this review, with contributions from SA, PB, and MK. JV and GR contributed to the conceptualization and reviewing of this review. All authors contributed to the article and approved the submitted version.

## Conflict of Interest

The authors declare that the research was conducted in the absence of any commercial or financial relationships that could be construed as a potential conflict of interest.
